# In GFP with high risk HPV-18E6 fusion protein expressed 293T and MCF-7 cells, the endogenous wild-type p53 could be transiently phosphorylated at multiple sites

**DOI:** 10.1186/1756-9966-27-35

**Published:** 2008-09-08

**Authors:** Lina Sun, Ge Zhang, Zongfang Li, Tusheng Song, Chen Huang, Lusheng Si

**Affiliations:** 1 Key Laboratory of Environment and Genes Related to Diseases of the Education Ministry, School of Medicine, Xi an Jiaotong University, Xi an, PR China; 2 State key Laboratory for Infectious Disease Prevention and Control, National Institute for Infectious Disease Control and Prevention, Chinese Center for Disease Control and Prevention, Beijing, PR China; 3 The second affiliated hospital, Xi an Jiaotong University, Xi an, PR China; 4 The first hospital of Shanxi Medical University, Taiyuan, PR China; 5 Key Laboratory of Environment and Genes Related to Diseases of the Education Ministry/Department of Genetics and Molecule Biology, School of Medicine, Xi an Jiaotong University, Xi an, PR China; 6 School of Life Science and Technology, Xi an Jiaotong University, Xi an, PR China

## Abstract

**Background:**

Infected cells recognize viral replication as a DNA damage stress and elicit the host surveillance mechanism to anti-virus infection. Modulation of the activity of tumor suppressor p53 is a key event in the replication of many viruses. They could manipulate p53 function through phosphorylation modification for their own purpose. But there is rarely research about p53 phosphorylation status in the context of HPV-E6. Therefore, we investigated whether p53 could be phosphorylated by HPV-E6.

**Methods:**

We used a mammalian green fluorescence protein (GFP) expression system to express HPV-18E6 with GFP fusion proteins (GFP-18E6) in wild-type (wt) p53 cell lines, such as 293T and MCF-7 cells to trace the traffic and subcellular location of E6 protein. By immunofluorescence technique and immunoblotting, we determined the positive phosphorylated sites of p53 and observed the distribution of phosphorylated p53 in the context of GFP-18E6.

**Results:**

GFP-18E6 was predominantly located in nuclei of wt p53 cell lines, and it could induce transient phosphorylation of p53 at multiple sites, such as Ser^15^, Ser^20^, and Ser^392^. All the three sites of phosphorylated p53s were localized in nuclei together with GFP-18E6.

**Conclusion:**

In GFP with high risk HPV-18E6 fusion protein expressed 293T and MCF-7 cells, the endogenous wt p53 could be transiently phosphorylated at multiple sites.

## Background

Human papillomaviruses (HPVs) are small double-stranded DNA viruses with a genome of approximately 8 kb [1,2]. Over 90% of human cervical carcinoma is associated with high risk mucosal HPVs, mainly the serotypes 18 and 16 [3]. The mechanisms underlying the actions of high risk HPVs leading to cancer have been studied extensively, and it was shown that the E6 and E7 proteins were the oncoproteins interacting with tumor suppressors p53 and pRb, respectively, and leading to infected-cell transformation and dysregulated proliferation [4,5]. Previous studies also showed that the principle activity of E6 was to target and degrade p53, therefore, p53's growth regulatory functions is abolished [6]. However, many authors reported the expression of E6 was not necessarily equated to a p53 null background [7-10]. Therefore, we hypothesized there might be other ways for E6 interaction with p53.

p53 is a very important tumor suppressor, it can be activated in response to DNA damage stresses [11-13]. Phosphorylation of p53 has been studied intensively and has been proposed to play a critical role in the stabilization and activation of p53 [14]. Infected cells recognize viral replication as a DNA damage stress and elicit the host surveillance mechanism to anti-virus infection [15]. The modulation of p53 function by phosphorylation seems to be a major antiviral defense mechanism employed by cells [16,17]. On the other hand, some viruses have evolved strategies such as reducing the phosphorylation of p53 for counteraction p53 activation. For example, Kaposi's sarcoma associated herpesvirus (KSHV) is associated with the pathogenesis of Kaposi's sarcoma, KSHV viral interferon regulatory factor1 (vIRF1) greatly reduced the level of serine 15 phosphorylation of p53, resulting in an decrease of p53 stability which could circumvent host growth surveillance and facilitate viral replication in infected cells [15]. But there is rarely research about p53 phosphorylation status in the context of HPV-E6.

## Methods

### Construction of expression vector

Full length HPV-18E6 sequence was amplified by PCR from HPV type 18 complete genome, and then cloned in frame within the C terminus of GFP at the Bgl II and EcoR I sites of the polylinker regions of the mammalian expression vector pGFP (Clontech, Palo Alto, CA), producing plasmid pGFP-18E6.

### Cell culture and transfection

The human embryonic 293T kidney cells and human breast adenocarcinoma MCF-7 cells were maintained in RPMI1640 medium (Gibco) supplemented with 10% fetal bovine serum (FBS) at 37°C in a humidified atmosphere of 5% CO_2_. Cells were seeded on glass coverslips in 12-well cell culture plates. The cells were transiently transfected with plasmid pGFP -18E6, pGFP overnight using Lipofectamine 2000 transfection reagent (Invitrogen, Carlsbad, Calif).

### Cell imaging by fluorescent microscope

The 293T and MCF-7 cells were grown on glass coverslips, transfected, and fixed with 4% paraformaldehyde for 10 min at room temperature, rehydrated three times with cold PBS, then stained with DAPI (4',6-Diamidin-2'-phenylindoldihydrochlorid) at 37°C in the dark for 10 min, rinsed again with PBS and mounted on slides. Cell images were collected with a Nikon fluorescent microscope at a magnification of ×400. Fluorescent images were analyzed using Nikon Software.

### Immunoblotting analysis

For each sample, 10^6 ^cells were collected by centrifugation (1000 × rpm for 5 min), washed once with ice cold PBS, and lysed in 100 μl RIPA buffer containing 50 mM Tris-HCl [pH 7.4], 150 mM NaCl, 1% NP-40, 0.5% sodium deoxycholate, 1 mM EDTA, 2.5 mM glycerophosphate, 1 mM PMSF, 10 mM NaF, and phosphatase inhibitor cocktail (Roche Diagnostics, Mannheim Germany). Protein concentration was determined using the BCA reagents (Pierce, Rockford, IL). Samples (30 μg) were analyzed on 12% SDS polyacrylamide gels, transferred to PVDF membranes (Invitrogen), and blocked for 1 h at room temperature with 5% non-fat milk in TBS buffer (20 mM Tris-HCl [pH 7.5], 0.5 M NaCl). The membranes were then incubated with the primary antibody overnight at 4°C. After three washes with TBS, the membranes were incubated with the secondary antibody for 30 min at room temperature. After three additional washes, the proteins were visualized by enhanced chemiluminescence (ECL) (Amersham Pharmacia, Piscataway, New Jersey, USA).

The following primary antibodies were used: anti-phospho-p53 Ser6, anti-phospho-p53 Ser9, anti-phospho-p53 Ser15, anti-phospho-p53 Ser20, anti-phospho-p53 Ser37, anti-phospho-p53 Ser46, anti-phospho-p53 Ser 392, anti-chk2 (Cell Signaling; dilution, 1:1000) and anti-ATM (sigma; dilution,1:1000).

### Immunocytochemistry

The cells were seeded on glass coverslips at a density of 1 × 105 cells/well. Then, they were transfected with plasmid pGFP -18E6 and pGFP overnight following standard procedures. After transfection, the cells were washed with PBS and fixed with 4% paraformaldehyde for 10 min at room temperature. They were then rehydrated three times with cold PBS, permeabilized with 1% Triton X-100 for 5 min on ice, and rinsed with PBS and blocked. The cells were incubated with primary antibodies overnight at 4°C. Subsequently, signal detection was performed using Cy3 -conjugated goat anti-rabbit IgG (Sigma; dilution, 1:200) in blocking solution for 30 min at room temperature in the dark. Then, the cells were washed three times with PBS and examined by confocal microscopy. Fluorescent images were analyzed using a Leica Confocal Software (Leica Microsystems).

### Statistics

All data were recorded as means ± standard deviation, and analyzed by the SPSS 11.0 software. Analysis of data was performed using one-way ANOVA for multiple comparisons. P values < 0.05 were considered statistically significant.

## Results and discussion

### GFP-18E6 was mainly located in nuclei

**Figure 1:**
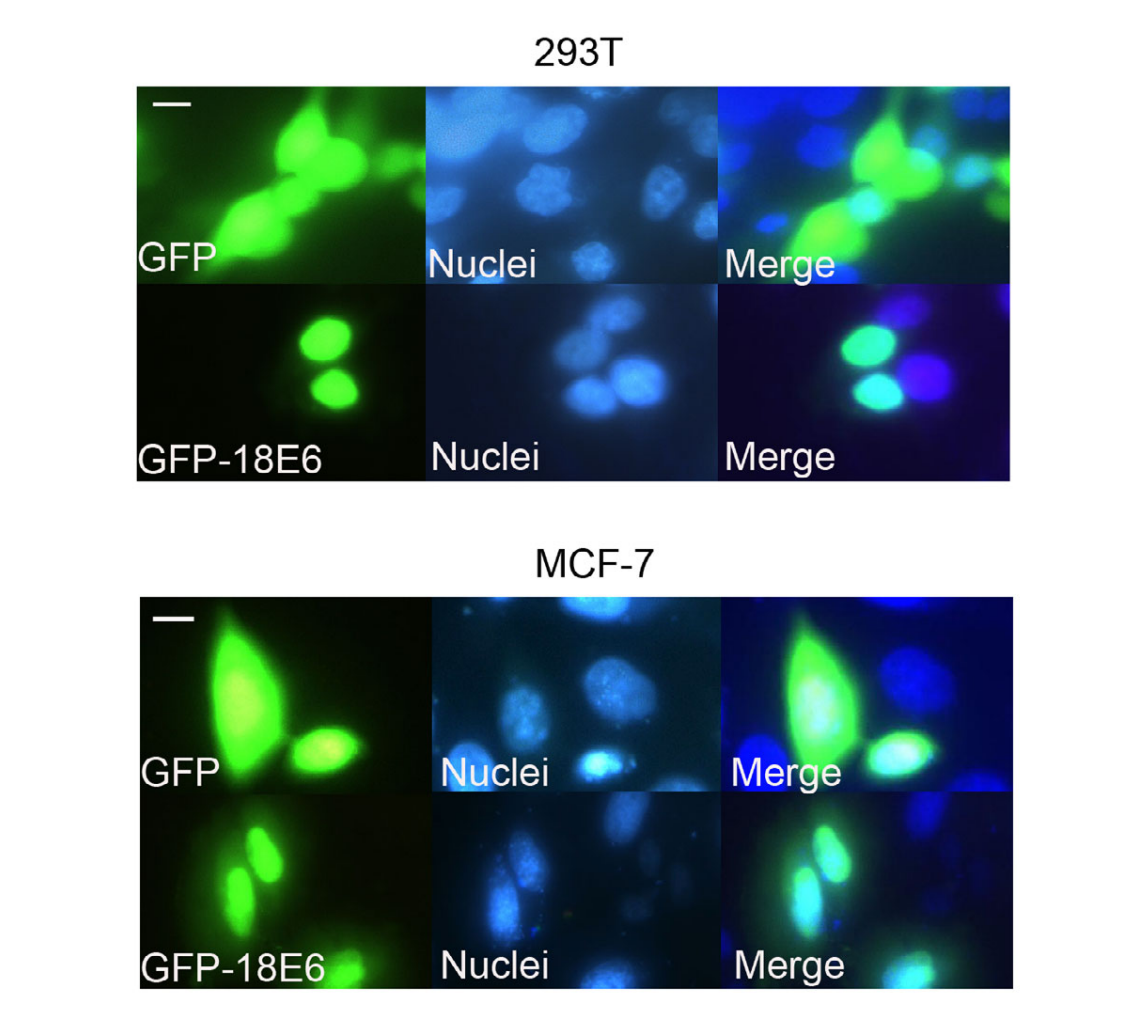
**GFP-18E6 is predominantly located in nuclei**. Representative photographs of 293T and MCF-7 cells at 21 h after transfection with GFP and GFP-18E6 expression plasmid. The green fluorescence is emitted by the cells transfected with pGFP and pGFP-18E6 respectively. The red is DAPI stained nuclei. Scale bar = 8 μm. The photographs are examined at 400× magnification by fluorescence microscope.

Viral E6 coding regions were inserted within the C terminus of the pGFP vector, producing plasmid pGFP-18E6. The plasmid pGFP-18E6 was transfected in 293T and MCF-7 cells, allowing E6 proteins to be expressed as GFP-18E6 fusion proteins. By fluorescence microscopy, we observed the subcellular location of GFP-18E6 and GFP in both cell lines. Because the E6 fusion proteins may have low or high expression levels at different times, and maybe this could affect the distribution of E6. Therefore, we dynamically observed the location and expression of proteins from 6 h to 72 h post-transfection. The results indicated that GFP-18E6 protein was expressed essentially in the nuclei from 6 h post-transfection. Its expression increased gradually, and reached its maximum expression level at 21 h (P < 0.001). Then, it decreased gradually and disappeared after 1 wk. During this whole period, no change was observed in protein location. As control, we observed the expression of GFP alone. It exhibited a diffused signal, and was present in both the nuclei and cytoplasm from 6 h to 1 wk post-transfection. In addition, its location did not change at any time. Representative photographs of the subcellular location of high risk GFP-18E6 and GFP were shown in Figure 1.

**Figure 2:**
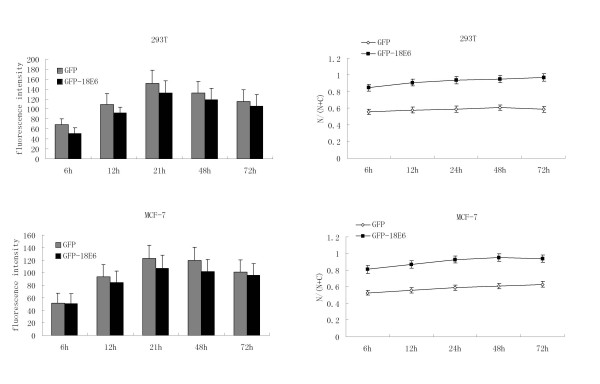
**Data analysis of GFP-18E6 level in 293T and MCF-7 cells**. The expression level of GFP and GFP-18E6 are examined by fluorescence intensity dynamically. One hundred cells are examined for each plasmid from 20 random fields. The N/(N+C) indicates fluorescence intensity ratio of GFP fusion protein in nuclei (N) to it in both nuclei and cytoplasm (N+C).

The analysis of relative fluorescence signal intensity of GFP-18E6 in 293T and MCF-7 cells was shown in Figure 2. We further studied the fluorescence intensity ratio of GFP fusion protein in nuclei (N) to it in both nuclei and cytoplasm (N+C) dynamically. In 6 h to 72 h post-transfection, E6 protein essentially located in nuclei and its value of N/(N+C) was increased from 80% to more than 90% gradually. As GFP control expressing cells, it was present in both nuclei and cytoplasm, and its value of N/(N+C) maintained in 50–60% during the whole period (Figure 2). Taken together, in the GFP with HPV-18E6 fusion protein expressing system, we observed GFP-18E6 was predominantly located in nuclei of 293T and MCF-7 cells.

### GFP-18E6 promoted multiple sites phosphorylation of p53 along with up-regulation of ATM and Chk2

**Figure 3:**
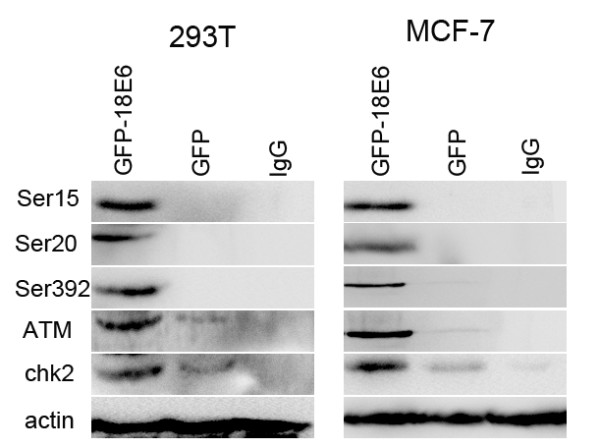
**HPV-18E6 promotes multiple sites phosphorylation of p53 along with up-regulation of ATM and Chk2**. The phosphorylated responses appear obviously at three sites: Ser^15^, Ser^20^, and Ser^392 ^of p53 along with the up-regulation of ATM and Chk2 at 24 h in GFP-18E6 expressing 293T and MCF-7 cells. IgG is an irrelative antibody used as the negative control. Data are normalized to β-actin and representative of three independent western blot analyses.

Because many viruses could manipulate p53 function through phosphorylation modification [16,17], we further investigated whether the wt p53 could be phosphorylated by GFP-18E6. We used antibodies for different sites that recognizing p53 only when it had been modified at these sites. By immunoblotting, we clearly observed phosphorylated p53 at 24 h post-transfected with pGFP-18E6 in both 293T and MCF-7 cells. The result indicated GFP-18E6 could induce p53 phosphorylation at three sites: Ser^15^, Ser^20^, and Ser^392^. As control GFP expressing cells, there was no phosphorylated p53 (Figure 3). The other sites of p53, such as Ser^6^, Ser^9^, Ser^37^, and Ser^46 ^were negative in both GFP-18E6 and GFP expressing cells (data not shown). Because the ataxia telangiectasia-mutated kinase (ATM) is critical for tansducing DNA damage signals to checkpoint control proteins [18,19], we next asked whether the phosphorylated p53 was associated with ATM activation. By immunoblotting, we observed the up-regulation of ATM and Chk2 (checkpoint kinase 2) in GFP-18E6 cells (Figure 3). Therefore, our study agreed with the activation of ATM resulted in phosphorylation or the activation of downstream checkpoint controls, including p53 and Chk2 [20].

### Co-localization of GFP-18E6 and phosphorylated p53 proteins

**Figure 4:**
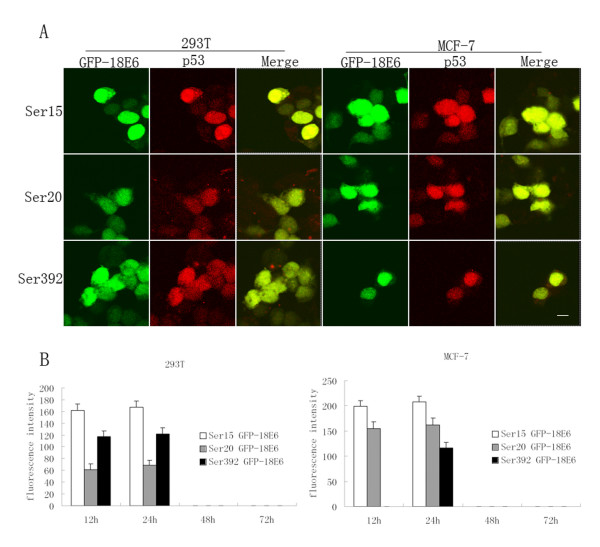
**Localization and expression level of phosphorylated p53 proteins**. (A) In 293T and MCF-7 cells, the phosphorylated p53s are located in nuclei together with GFP-18E6. Green fluorescence indicates the protein of GFP, and GFP-18E6 expressed by the transfected cells, Red fluorescence indicates phosphorylated p53 proteins, which are labelled with phosphorylated anti-p53 antibodies plus anti-rabbit-Cy3 secondary antibody. The photographs are examined at 400× magnification by confocal microscopy. Scale bar = 8 μm. The results shown are representative of three independent experiments. (B) Level of phosphorylated p53 in the context of GFP-18E6 from 12 h to 72 h. The data of phosphorylated p53s level are examined by fluorescence intensity. 100 cells are examined for each phosphorylated site of p53 from 20× random fields.

Because high risk HPV-E6 can target and interact with p53 [21], we suspected that the GFP-18E6 and phosphorylated p53s might locate together. By immunocytochemistry staining, we observed phosphorylated p53 proteins at three sites, including Ser^15^, Ser^20 ^and Ser^392^, which were all highly expressed at 24 h post-transfected with pGFP-18E6 in 293T and MCF-7 cells. This was consistent with our results by immunoblotting analysis as noted above. Furthermore, we observed the phosphorylated p53s were located in nuclei together with GFP-18E6. Figure 4A shows representative photographs of the co-localization of GFP-18E6 and phosphorylated p53 proteins. Taken together, the three sites of phosphorylated p53s were essentially located in nuclei together with GFP-18E6.

### Level of phosphorylated p53 along with time course

Since the expression of GFP-18E6 was associated with time course, we next determined the three sites of phosphorylated p53 level in 293T and MCF-7 cells from 12 h to 72 h post-transfection dynamically. For GFP-18E6 expressing cells, the Ser^15^, Ser^20 ^of p53 were firstly detected at 12 h post-transfection and increased gradually, significant accumulation was observed at 24 h (P < 0.001). The expression level of Ser^15 ^was higher than Ser^20 ^at the same time point. It should be noted that phosphorylation of Ser^392 ^was not present at 12 h in GFP-18E6 transfected MCF-7 cells, whereas it was highly expressed in GFP-18E6 transfected 293T at the same time. In both cells, the Ser^392 ^reached highest level at 24 h post-transfection (P < 0.001). From 48 h to 72 h post-transfection, the three sites of phosphorylated p53s were not detected. As GFP control expressing cells, there was not phosphorylated p53 at Ser^15^, Ser^20^, and Ser^392 ^during the whole period (Figure 4B). Thus, in 12 h to 24 h expression of GFP-18E6 there was a short term activation of p53 by phosphorylating modification. Because activation of p53 can also be modulated at the transcription level [22,23], we next asked whether the mRNA level of p53 was increased by the expression of GFP-18E6. By reverse transcriptase (RT)-PCR, we clearly observed the mRNA of p53 was not changed in GFP-18E6 expressing cells (data not shown). This agreed with the mRNA level of p53 was stable in the context of HPV-E6 [24,25].

Phosphorylation of p53 at Ser^15 ^and Ser^20 ^were the earliest response to E6 expression. It is general believed Ser^15 ^phosphorylation of p53 occurs rapidly in response to DNA damage and appears to represent a 'priming event' for the subsequent series of modifications [26]. Because phosphorylation of Ser15 induced by ATM/ATR (ATM-and-Rad3-related) results in dissociation of p53 from its negative regulator mdm-2, it has been suggested that the primary effect of phosphorylation of p53 at Ser^15 ^is to increase p53 level [27]. The Ser^20 ^is also critical for stabilizing of p53. Recent studies have demonstrated that Ser^20 ^on p53 is phosphorylated by Chk1 (checkpoint kinase 1) or Chk2, enhancing its tetramerization, stability, and activity in response to DNA damage [28]. In fact, phosphorylated sites at the Ser^15 ^and Ser^20 ^residues lie right under the binding pocket of mdm-2, which could disrupt the binding with mdm-2, resulting stabilization of p53 [29]. In the present study, the level of phosphorylation of p53 at Ser^15 ^was clearly higher than Ser^20^. It's probably because Ser^15 ^phosphorylation of p53 was a more important target than Ser^20 ^in the context of HPV-18E6. This was consistent with some data reported that removing Ser^15 ^can abrogate phosphorylation at Ser^20 ^[30]. For phosphorylation of p53 at Ser^392^, it was even not same for both cells. In 293T cells, phosphorylation of p53 at Ser^392 ^appeared earlier and higher than MCF-7 cells. Thus, the different responses of Ser^392 ^maybe due to varied sensitivity of cells. Authors reported phosphorylation of p53 at Ser^392 ^was an early response to a wide range of stress-inducing conditions. Ser^392 ^is phosphorylated by the protein kinase CK2 after both UV and ionizing radiation treatment [31]. It has been shown to enable the transcriptional activation of the p53 protein in vitro and also seems to be important for p53-mediated transactivation in vivo [32,33]. Therefore, the phorsphorylation of p53 at Ser^15^, Ser^20^, and Ser^392 ^could stabilize and activate p53, which ultimately induces the irreversible cell cycle arrest and apoptosis in response of DNA damage stress [30].

It has been proved the tumor suppressor p53 could induce cell cycle arrest or apoptosis in response to stresses, such as UV radiation, DNA damage, hypoxia or virus infection [11,12]. Previous also studies showed E6 could target and degrade p53, which in turn inhibited apoptosis [4]. In present study, we clearly observed the phosphorylating modification of p53 in the early stage of HPV-18E6 expressing. The phosphorylation of p53 could induce apoptosis or cell cycle arrest in response to DNA damage stresses [29-31]. Regulation of p53 phosphorylation has also been shown to be induced by many viruses, such as, Africa swine fever virus (ASFV), the p53 in host cell is stabilized by phosphorylation at Ser^392 ^and is located in the nuclei. During infection, the phosphorylated p53 is functionally active to induce apoptosis along with the expression of p21 and mdm2 [16]. The Epstein-Barr virus (EBV) can activate p53 through phosphorylated modification at Ser^15^, Ser^20^, and Ser^392 ^modulated by its oncogenic protein LMP1. Additionally, the phosphorylated p53s were associated with MAPK (mitogen-activated protein kinase) and the activation of MAPK kinase could target the transcription factors to anti-virus infection [17]. Thus, our result agreed with Shin and others, who reported infected cells recognized viral replication as a DNA damage stress and elicit the host surveillance mechanism to anti-virus infection [15]. On the other hand, malignant transformation usually takes a long term, where it is important that oncogene E6 integrated in the genome of host and degraded p53 [34]. It was easy to understand, in the present study, we constructed a short transient expression system, the over-expression of E6 can partly mimic the large infection of HPV in the early stage, where the oncogene E6 is not integrated in the genome of host. With over-expressed exotic DNA of E6, the host cells might clean them up through activating apoptosis pathway. This also agreed with authors, who reported compared to the prevalence of HPV infections in the general population, the number of lesions that progress to cancer is very low [35]. The cells infection with high risk HPV might take a self limited process through apoptosis mechanism without progressing to cancer. Thus, the present study gave us an implication that giving the patient correct treatment on the early stage of HPV infection, which will be helpful not to progress to cancer.

## Conclusion

In conclusion, the present study provided a new pattern of interaction between HPV-18E6 and p53. That was, GFP-18E6 could transiently induce p53 phosphorylation at three sites, Ser^15^, Ser^20^, and Ser^392 ^in both 293T and MCF-7 cells by the activation of ATM and/or Chk2 pathway.

## Competing interests

The authors declare that they have no competing interests.

## Authors' contributions

CH, LSS and TS participated in its design, discussed the results, and helped to draft the manuscript. GZ and ZL carried out the immunofluorescence microscopy. LS conceived of the study, participated in its design, carried out experiments, and wrote the manuscript. All authors read and approved the final manuscript.
